# Importance of the Septal Gingival Margins

**Published:** 1919-02

**Authors:** H. E. Friesell


### IMPORTANCE OF THE SEPTAL GINGIVAL MARGINS

BY H. E. FRIESELL, D.D.S.

That portion of the gum tissue which lies to the occlusal of the crest of the alveolar process is called the gingiva. It is subdivided into the body of the gingiva, the free gingiva and the septal gingiva. The body consists of that portion of the gingiva which lies between the crest of the alveolar process and the gingival line.

The free gingiva and the septal gingiva are attached to the body of the gingiva and form the free flap of gum tissue which covers certain portions of the enamel. The free gingiva occupying the interproximal spaces is called the septal gingiva.

The filling of the septal space with gingiva and the health of that septal gingiva depend primarily upon the proper size and form of the interproximal space. It will be noted that the interproximal spaces between the anterior teeth are necessarily much narrower than are those between the posterior teeth; but Nature has provided for this condition by extending the proximal curvature of the gingival line much further toward the occlusal on the anterior teeth.

The free gingiva hugs the teeth tightly but is not attached thereto. As it approaches its occlusal extremity it becomes thinned to a very fine edge, which serves, so long as the tissue remains healthy, to prevent the ingress of food debris, etc., into the sub-gingival space.

The septal gingiva is in the form of an arch, or wedge, with its crest or apex towards the occlusal and just beneath the contact point. It slopes off in two inclines towards the gingival, the shorter and steeper slope being toward the buccal, and the longer and more gentle slope being through the lingual embrasure. Between the anterior teeth the slopes are nearly equal.

The tooth is held in place by the peridental membrane; when this membrane becomes diseased the tooth suffers proportionately in loss of function; when the peridental membrane is lost the tooth goes with it. When the peridental membrane becomes exposed to the secretions and the flora of the mouth it readily becomes diseased. It does not endure exposure to mouth conditions.

The gingiva therefore serves as a covering and protection not only to the crest of the alveolar process, but to the peridental membrane as well. A healthy peridental membrane is absolutely essential to the retention of the tooth, and as the protection of the peridental membrane depends so largely upon a healthy gingiva, the care of the latter tissue and the maintenance of its health becomes paramount in dental practice.

Injury to the gingiva by the impaction of food, the encroachment of calculary deposits, the irritation of dental appliances, or by the careless or ignorant use of instruments must be carefully guarded against.

The impaction of food, following the ravages of caries or unskillful dentistry is the most prolific cause of injury to the gingiva and peridental membrane. Tooth form as it exists originally, or as it is properly restored will fully protect these tissues against such injury.

The height of contour on the axial surfaces of the teeth causes the food to be shunted off and strike the gingiva at a tangent instead of directly, as happens when the height of contour is lost.

The contact points hold the teeth in their proper relationship to each other occlusally; maintain the proper mesio-distal width of the inter-proximal space; and protect the crest of the arch of the septal gingiva. They prevent the food from passing directly through and crushing the crest of the arch, and divert it bucally and lingually into the embrasures, where it strikes the inclines or slopes of the septal gingiva at a tangent and passes harmlessly into the buccal or lingual cavity, to be returned to the occlusal surface by the tongue or cheeks until mastication is complete.

The mesial and distal marginal ridges serve as rims to keep as much of the food as possible on the occlusal surface where it can be crushed by the occluding teeth. Their proximal slopes help to give proper approach and contour to the contact point and height of contour, and the form of the marginal ridges, contact points, and height of contour combined serves to direct the food that passes into the proximal spaces in such a way that it will do no injury to the investing tissues.

If the contact be too broad bucco-lingually it will form a line contact instead of a point; will diminish the embrasures and prevent the scouring of much of the proximal enamel. If the contact be too broad gingivally as well as laterally, it will become a surface contact, food fibres will be caught and held between the teeth, wedging them àpart, and as other fibres accumulate, the whole mass will crush against the septal crest, flattening it and leaving a space between the teeth to bcome filled with food debris and give rise to injury of the tooth or gingiva through fermentative or putrefactive changes.

Sometimes in cases of proximal impaction of the food from faulty contacts we find the septal tissue not only flattened bucco-lingually, which permits food to strike it directly instead of at a tangent, but even depressed in the center, forming a pocket. Inflammation results and the gingiva in the buccal and lingual embrasures becomes swollen and hypertrophied; suppuration occasionally results and a gingival abscess develops, which not infrequently discharges on the buccal or lingual surface of the gum, and simulates the opening of a sinus from an alveolar abscess.

If the proper form of a tooth be maintained in its marginal ridges, height of contour and contact points, the most prolific cause of peridental disease will be eliminated. The dentist who makes himself familiar with tooth form and develops the skill to restore it, or maintain it, may be safely trusted to work no injury to the investing tissues of the teeth through the placing of crude appliances, or careless instrumentation. I doubt whether any other kind of a dentist should be permitted to operate in the human mouth.—Extract from paper printed in the *Dental Items of Interest* for January 1919.

